# Age‐ and sex‐specific reference intervals for superoxide dismutase enzyme and several minerals in a healthy adult cohort

**DOI:** 10.1002/jcla.23897

**Published:** 2021-07-17

**Authors:** Hamideh Ghazizadeh, Mary Kathryn Bohn, Mahdiyeh Yaghooti‐Khorasani, Atieh Kamel Khodabandeh, Reza Zare‐Feyzabadi, Ameneh Timar, Maryam Mohammadi‐Bajgiran, Mohammad Reza Oladi, Mohadeseh Rohban, Habibollah Esmaily, Gordon A. Ferns, Khosrow Adeli, Majid Ghayour‐Mobarhan

**Affiliations:** ^1^ Student Research Committee Mashhad University of Medical Sciences Mashhad Iran; ^2^ Metabolic Syndrome Research Center Mashhad University of Medical Sciences Mashhad Iran; ^3^ International UNESCO Center for Health‐Related Basic Sciences and Human Nutrition Mashhad University of Medical Sciences Mashhad Iran; ^4^ CALIPER Program Division of Clinical Biochemistry Pediatric Laboratory Medicine The Hospital for Sick Children Toronto ON Canada; ^5^ Department of Laboratory Medicine & Pathobiology University of Toronto Toronto ON Canada; ^6^ Social Determinants of Health Research Center Mashhad University of Medical sciences Mashhad Iran; ^7^ Brighton & Sussex Medical School Division of Medical Education Brighton UK

**Keywords:** macro‐mineral, micro‐mineral, reference interval, superoxide dismutase

## Abstract

**Introduction:**

The aim of this study was to establish RIs for clinically important markers including superoxide dismutase (SOD), serum copper, zinc, calcium, magnesium, and phosphate in a cohort of healthy Iranian adults.

**Materials:**

A subsample from MASHAD cohort study was used to assess serum SOD, copper, zinc, calcium, magnesium and phosphate. Serum SOD was measured according to its inhibitory potential of pyrogallol oxidation. Micro‐ and macro‐minerals were measured using flame atomic absorption spectrometry and a BT3000 autoanalyzer, respectively. Sex‐ and age‐specific RIs were then calculated based on CLSI Ep28‐A3 guidelines.

**Results:**

Reference value distributions for studied parameters did not demonstrate any age‐specific differences that were statistically significant. In addition, sex partitioning was not required for all parameters, apart from serum magnesium, which showed a wider range in females (0.81–1.26 mg/dl) compared with males (0.82–1.23 mg/dl).

**Conclusion:**

The RIs established in this study can be expected to improve mineral assessment and clinical decision‐making in the Iranian adult population.

## Graphical abstract

The aim of this study was to establish RIs for clinically important markers including superoxide dismutase (SOD) and serum copper, zinc, calcium, magnesium and phosphate in a cohort of healthy Iranian adults. Sex‐ and age‐specific RIs were then calculated based on CLSI Ep28‐A3 guidelines. Age and sex partitioning were not required for all parameters, apart from serum magnesium, which showed a wider range in females (0.81–1.26 mg/dl) compared to males (0.82–1.23 mg/dl).

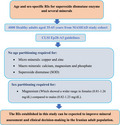

## 1 INTRODUCTION

Accurate disease diagnosis and monitoring relies heavily on the accuracy of laboratory test results as well as the reference intervals (RIs) reported for test interpretation.^1^ RIs can be defined as health‐associated benchmarks, commonly calculated as the central 95% of laboratory test values observed each in a healthy reference population.^2^, ^3^, ^4^ While the same RIs are often widely applied across different geographical regions, genetic and environmental differences can influence laboratory test results.^5^, ^6^ In addition, age, sex, diet, and lifestyle can influence disease manifestation and also has a major impact on circulating biomarker levels, in both healthy and unhealthy subjects. For instance, serum concentrations of certain fatty acids or plasma phospholipids may reflect medium‐term intake of various foods and can related to habitual intake of specific dietary fats or fatty acids in different populations.^2^, ^3^, ^7^ Therefore, specific RIs for each population may be required.

The International Federation of Clinical Chemistry (IFCC) has published many reports recommending each laboratory establishes specific RIs based on the age, sex, and ethnicity of its population, or validates a pre‐existing RI given common resource limitations.^8^ These recommendations particularly relate to minerals which are likely to differ based on the nutritional status of different geographical regions.^9^, ^10^ Minerals can be divided into two categories: macro‐minerals and micro‐minerals.^11^ Calcium, magnesium, and phosphate are considered macro‐minerals which are needed in greater amounts compared with micro‐minerals like zinc and copper.^11^ Minerals play important roles in both physiological and pathological processes, and evaluation of these factors is important to assess health status. However, careful interpretation of test results is essential as several factors such as sex, age of population, geographical area, lifestyle, dietary intake, and diet/drinking water can influence serum levels of minerals.^9^, ^10^, ^12^

The physiological roles of minerals are diverse. For example, zinc and copper have effects on growth, neuronal development, immunity, bone and blood health, and cell apoptosis,^13^, ^14^ while magnesium plays an important role in carbohydrate metabolism, acting as a cofactor in several enzymatic reactions.^15^ Calcium and phosphate form the main contents of the bone structure^16^ in the form of hydroxyapatite [Ca_10_(PO_4_)_6_(OH)_2_] crystals,^17^ playing a role in the formation of the bone by their effects on parathyroid hormone (PTH) and 1,25‐dihydroxyvitamin D.^18^ Finally, copper and zinc are involved in the structure of SOD, an enzyme with the role of reducing oxidative stress by converting the superoxide to oxygen and hydrogen peroxide.^19^ Copper and zinc form the part of the active site of this enzyme.^20^

Besides the specific roles of each of these minerals, it has been reported that the concentration of calcium, phosphate, magnesium, zinc copper, and SOD can act as biomarkers for several clinical conditions. For example, these minerals have been shown to differ in sickle cell anemic patients when compared with healthy controls, as a result of altered hemoglobin and production of reactive oxygen species (ROS).^21^ In addition, it has been established that SOD activity is enhanced in Hashimoto's thyroiditis due to elevated oxidative status. Hashimoto's thyroiditis has also been shown to be associated with hypomagnesaemia, hyperphosphatemia, and hypocalcemia due to the influence of thyroid hormones on the reabsorption of magnesium, phosphate, and calcium.^22^ In addition, it has been shown that plasma levels of SOD could be a useful clinical biomarker of oxidative stress in patients with end‐stage renal disease^23^ and might be considered in the identification of gastric adenocarcinoma from healthy control tissue.^24^ The metabolism of calcium, phosphate, and magnesium can also be altered in chronic kidney disease,^25^ a disease which cause vascular calcification because of the oxidative stress process.^26^ Also, concentration of calcium and phosphate is important for assessing skeletal function.^16^

Previous findings from other regions suggest that population‐specific RIs for each of these parameters are needed.^2^, ^27^, ^28^, ^29^, ^30^, ^31^ This study aims to determine age‐ and sex‐specific RIs for a specific region (northeastern of Iran) for calcium, phosphate, magnesium, zinc, copper, and SOD in middle‐aged population (35–65 years).

## 2 METHODS

### 2.1 Study population

The MASHAD cohort study was designed in 2010 and is currently ongoing. In this study, 9,704 participants were recruited and provided informed written consent. In the current study, RIs for macro‐ and micro‐minerals were determined by using available data from 1,000 and 4,040 men and women aged 35–65 years, respectively. Subjects with a diagnosis of cardiovascular disease, diabetes, hypertension, endocrine abnormalities, liver and kidney diseases, cancer and chemotherapy, major surgery, hepatitis, hematology disorders, and any chronic and/or acute health conditions which were professionally diagnosed, or who took prescribed medications, were excluded. Additionally, participants with high sensitivity C‐reactive protein (hs‐CRP) concentrations >10 mg/L were excluded. Furthermore, subjects who recently had surgery and blood transfusion were excluded. All health‐related information such as history of disease diagnosis and medication use was collected by healthcare professionals. After applying the exclusion criteria, missing data for some participants led to a difference in the sample size for each biomarker. The study was approved by the Human Research Ethics Committee of Mashhad University of Medical Sciences (MUMS).

### 2.2 Laboratory evaluation

Blood samples for all subjects were collected in Vacutainer^®^ tubes after 14‐h overnight fast. Following collection, samples were centrifuged at 5,000 *g* for 15 min at 4°C and then serum samples were frozen at −80°C for future analysis. Flame atomic absorption spectrometry was used to measure serum copper and zinc.^32^ The accuracy of this method was measured as 93% ± 4.8% and 95% ± 3.75%. The accuracy was evaluated based on the zinc and copper standard curves by utilizing zinc and copper standards (Merck and Co. Pharmaceutical Company) through evaluating the confirmed reference material (Merck KGaA 64271 Darmstadt, Germany) comprising known values (1,000 ± 2 mg/L).^32^ The activity of SOD was determined as described by Torkanlou.^33^ Briefly, a 96‐well‐plate microassay was utilized according to the inhibitory potential of pyrogallol oxidation, which was adapted for SOD, and the absorption was measured at 405 nm. Calcium and phosphate were measured by using a photometric method and Pars Azmoon kits (Tehran, Iran), on an autoanalyzer BT3000.^34^ Specifically, the amount of calcium was measured based on the intensity of the purple color generated by the interaction between calcium and Cresolphthalein Complexone. Phosphate was measured based on the intensity of the color induced by the interaction between phosphate and ammonium molybdate and sulfuric acid.^34^ Magnesium also was assessed by using the xylidyl blue photometric method and the BT3000 autoanalyzer (Biotechnica, Rome, Italy) (Pars Azmoon kit [Tehran, Iran]).

### 2.3 Statistical analysis

Age‐ and sex‐specific partitioning was assessed visually and confirmed statistically by using the Harris and Boyd method.^35^ Extreme outliers were manually removed based on visually inspection. Additional statistical outliers were then identified using Tukey's method,^36^ if normally distributed, and the adjusted Tukey method, if skewed.^37^ Following outlier removal, the nonparametric rank method was utilized to calculate the RIs, defined as the central 95% (2.5–97.5%), in alignment with CLSI Ep28‐A3 guidelines. Corresponding 90% confidence intervals (CIs) were also calculated for both the upper and lower reference limit intervals (CIs).^3^

## 3 RESULTS

**FIGURE 1:**
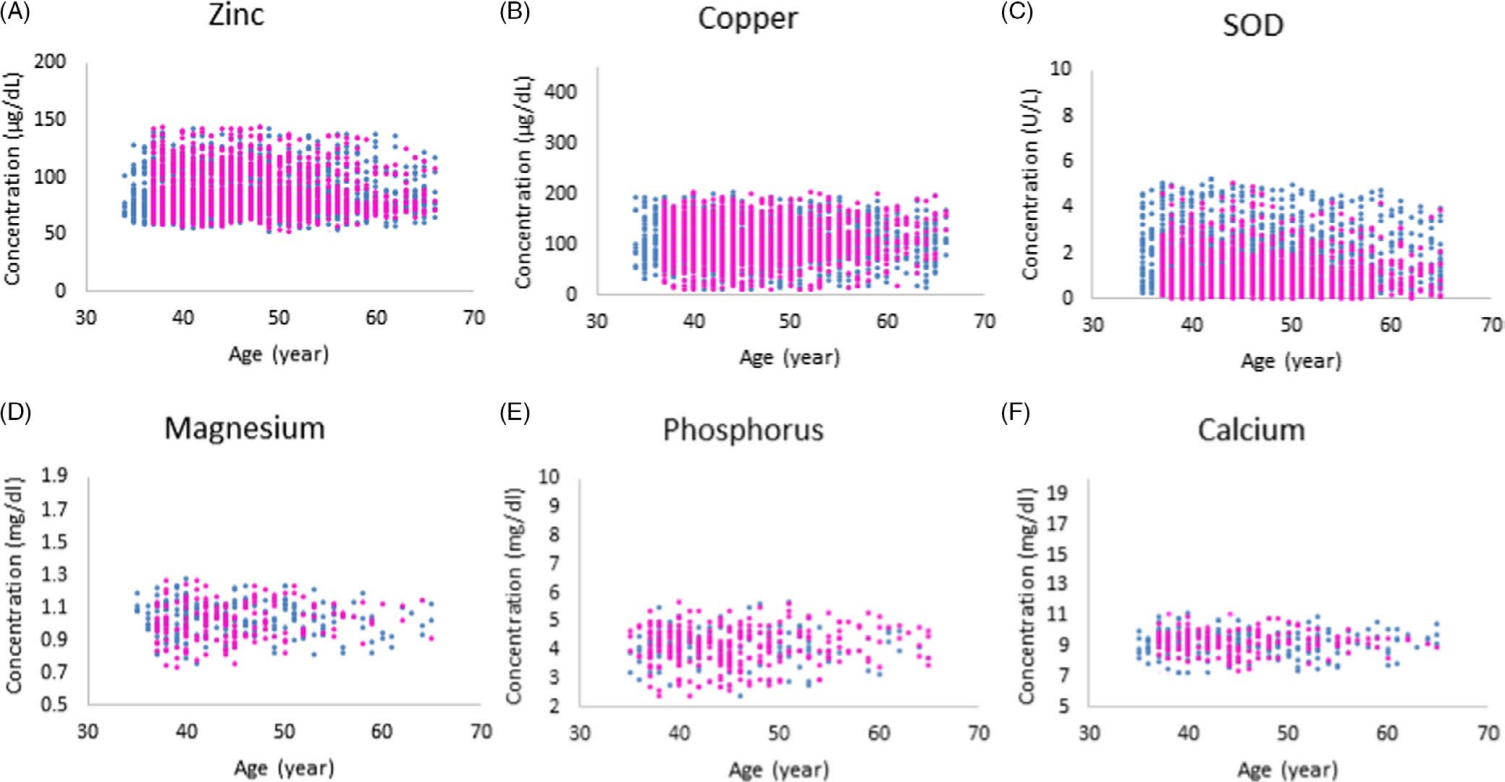
Scatterplot distributions for serum zinc (A), copper (B), superoxide dismutase (SOD) (C), magnesium (D), phosphorus (E), and calcium (F) in total population. Females and males are shown by pink and blue circles, respectively

Reference intervals for magnesium, calcium, and phosphate were determined by using available data on 1,000 men (445) and women (433) and for zinc, copper, and SOD in a population sample of 4,040 men (2,112) and women (1,928) aged 35–65 years from the MASHAD cohort study. After applying the exclusion criteria and removing outliers and missing data, the sample size for each biomarker differed (Table 1). Established RIs for each parameter are defined in Table 1 and associated scatterplots are shown in Figure 1, depicting reference value distributions by age and sex.

**TABLE 1:** Reference intervals for minerals based on data from Mashhad Stroke and Heart Atherosclerotic Disorder (MASHAD) study, Mashhad, Iran

Total population (*N* = 4,040)						
Serum variables	Sample size (*n*)	Lower limit	Upper limit	Lower confidence interval	Upper confidence interval	The intra‐ and inter‐assay coefficient of variation (CV%)
Calcium (Mg/dl)	399	7.5	10.5	7.3–7.7	10.5–10.7	0.2 & 0.2
Phosphorus (Mg/dl)	418	2.8	5.4	2.7–2.9	5.3–5.6	0.2 & 0.2
Magnesium (Mg/dl)						
M	219	0.82	1.23	0.79–0.84	1.21–1.25	0.2 & 0.2
F	170	0.81	1.26	0.78–0.84	1.17–1.29	
Zinc (µg/dl)	3,085	68.6	135.0	68.2–69.2	133.3–140.5	1.5 ± 0.2 & 2.6 ± 0.4
Copper (µg/dl)	3,293	30.00	181.65	26–31	179.3–187.3	1.3 ± 0.12 & 2.11 ± 0.32
SOD (U/L)	2,641	0.40	4.99	0.40–0.40	4.79–5.12	‐

Abbreviations: F, female; M, male; SOD, superoxide dismutase.

For all studied parameters, no statistically significant age‐specific differences were noted. Thus, only one age partition was required for all parameters (35–65 years). Magnesium was the only parameter that demonstrated statistically significant sex‐specific differences, although the observed difference was minimal. Specifically, the established RIs for magnesium were wider in women (0.81–1.26 mg/dl) compared with males (0.82–1.23 mg/dl).

## 4 DISCUSSION

We have determined age‐ and sex‐specific RIs for five serum minerals and SOD enzyme levels in a healthy adult sample of the Iranian population from Mashhad city. RIs were calculated by applying a statistical methodology that has been previously described by the Canadian Laboratory Initiative on Pediatric Reference Intervals (CALIPER)^2^ and recommended by CLSI Ep28‐A3 guidelines.^3^ A summary comparison of our results to the existent literature is also present in Table 2.

**TABLE 2:** Comparison of RIs of MASHAD study with other studies

Serum variables	RI of other countries			RI of Iran
Calcium (Mg/dl)	Canada^38^ ^a^	Caxias do Sul^4^ ^c^	India^31^ ^b^	MASHAD study
	CLSI/2.5–97.5% M: 9.1–10.4 (20–39 YO); 9.0–10.2 (40–79 YO) F: 9.0–10.1(20–39 YO); 9.0–10.2 (40–79 YO)	CLSI/2.5–97.5% (18–66 YO) M: 8.0–10.7 F: 8.0–10.3	CLSI/2.5–97.5% (18–56 YO) 8.8–10.6	CLSI/2.5–97.5% (35–65 YO) 7.5–10.50
Phosphorus (Mg/dl)	Canada^38^ ^a^	Botswana^30^ ^d^	India^31^ ^b^	MASHAD study
	CLSI/2.5–97.5% M: 2.9–4.7 (16–47 YO); 2.8–4.7 (48–79 YO) F: 2.9–4.7 (16–47 YO); 3.1–4.8 (48–79 YO)	CLSI/2.5–97.5% (18–39 YO) 2.5–4.5	CLSI/2.5–97.5% (18–56 YO) 3.0–4.8	CLSI/2.5–97.5% (35–65 YO) 2.8–5.4
Magnesium (Mg/dl)	United states^41^ ^e^	Canada^42^ ^f^	India^31^ ^b^	MASHAD study
	CLSI/2.5–97.5% (18–74 YO) 1.8–2.3	CLSI/2.5–97.5% (20–57 YO) M: 1.06–1.45 F: 1.09–1.38	CLSI/2.5–97.5% (18–56 YO) 1.8–2.9	CLSI/2.5–97.5% (35–65 YO) M: 0.82–1.23 F: 0.81–1.26
Zinc (µg/dl)	Nigeria^48^ ^g^	Greece^49^ ^g^	Germany^29^ ^h^	MASHAD study
	CLSI/2.5–97.5% (18–56 YO) 62.04–134.29	CLSI/2.5–97.5% (18–60 YO) M: 39–150 F: 44–149	CLSI/2.5–97.5% (22–75 YO) 60.80–151.02	CLSI/2.5–97.5% (35–65 YO) 68.60–134.97
				Tehran^28^ ^g^
				(20–90 YO) M: 62.9–206.5 F: 58.2–198.5
Copper (µg/dl)	Nigeria^48^ ^g^	Greece^49^ ^g^	Germany^29^ ^h^	MASHAD study
	CLSI/2.5–97.5% (18–56 YO) 88.06–177.72	CLSI/2.5–97.5% (18–60 YO) M: 40–202 F:67–235	CLSI/2.5–97.5% (22–75 YO) 58.45–202.69	CLSI/2.5–97.5% (35–65 YO) 30.00–181.65
SOD (U/L)	Macedonia^27^ ^i^			MASHAD study
	CLSI/2.5–97.5% (18–59 YO) 0.12–0.33			CLSI/2.5–97.5% (35–65 YO) 4.00–4.99

Note

The assay methodology used for each study: ^a^The Vitros 5,600 FS analyzer (Ortho Clinical Diagnostics), CLSI C28‐A3 guidelines^38^; ^b^Olympus AU 400 Chemistry auto analyzer (Olympus Optical, Japan), CLSI C28‐A2 guidelines^31^; ^c^CLSI C28‐A3 guidelines^4^; ^d^NCCLS guideline^30^; ^e^atomic absorption by Perkin‐Elmer instrument^41^; ^f^automated potentiometric analyzer (the NOVA 8 electrolyte analyzer [NOVA Biomedical, Mississauga, ON, Canada]), NCCLS document C28‐A^42^; ^g^flame atomic absorption spectrometry^28^, ^48^, ^49^; ^h^electrothermal atomic absorption spectrophotometry^29^; ^i^Gaussian curve.^27^

Abbreviations: F, female; M, male; SOD, superoxide dismutase.

### 4.1 RIs for macro‐minerals

In this study, RIs for macro‐minerals (ie, calcium, phosphate, and magnesium) were evaluated. Previous studies have recommended age‐ and sex‐specific RIs for macro‐minerals and are discussed below.

Previously established RIs for calcium are reported in Table 2 for men and women in Canada,^38^ India,^31^ and the Caxias do Sul population.^4^ As seen, partitioning based on sex was applied in the Canadian and Caxias do Sul studies, though it was not necessary for the Indian population, as was the case in our Iranian population. Statistically significant age differences were also found in calcium concentrations in the Canadian study, likely due to robust sample size. The quantitative reference limits established for calcium across studies were fairly concordant, although an increased lower reference limit was reported in the Canadian and Indian studies when compared with our results.

Reference intervals reported for phosphate are also shown in Table 2 and compared with studies in men and women living in Canada,^38^ Botswana,^30^ and India.^31^ While each study assessed a slightly different age range, the need for sex and age partitioning was only reported in Canadians. The established upper reference limit in the current study was higher than all previous reports, while, the established lower reference limit was similar to that of other studies. Notably, these discrepancies may be related to study population characteristics, including the inclusion of adults younger than 35 years old in most comparative studies. Both serum calcium and phosphate are known to be affected by PTH and vitamin D concentrations.^18^ Thus, an assessment and comparison of PTH and vitamin D reference value distributions for each of these regions is necessary. In addition, kidney function, evaluated by creatinine (Cr) level and glomerular filtration rate (GFR),^39^ influences the levels of calcium and phosphate.^40^ Comparing the Cr levels and GFR between our population and others may also be useful in identifying the need for separate RIs. Additionally, the analytical method of measurement for each study can influence the reported RIs. For instance, the Vitros 5600 FS analyzer (Ortho Clinical Diagnostics) was used in Canadian population^38^ and the Olympus AU 400 Chemistry auto analyzer (Olympus Optical, Japan) was used in Indian study.^31^

Previous reported RIs for magnesium are also included in Table 2, for populations from India,^31^ United States,^41^ and Ottawa (Canada).^42^ Statistically significant sex differences in magnesium concentrations were only reported in the Canadian study, in alignment with our findings. However, a wider RI was reported for males in the Canadian study, in contrast to our findings that showed a wider range in females (0.81–1.26 mg/dl) compared with males (0.82–1.23 mg/dl). For all studies, no age‐related changes were noted. It is important to note that the RI for magnesium in our population (M: 0.82–1.23, F: 0.81–1.26) is much lower than the globally established cutoff for this element, as the concentration of magnesium ions <1.7 mg/dl is considered as hypomagnesemia and >2.5 mg/dl is defined as hypermagnesemia.^43^ The prevalence of hypomagnesaemia (<1.7 mg/dl) in 1,558 subjects of Tehran (a city of Iran) was defined 4.6% in a previous study.^44^ It is possible that the used method for magnesium ion detection affects the results, as the analytical measurements varied between studies.^45^ Specifically, methods of ionized magnesium measurement were atomic absorption by Perkin‐Elmer instrument in United States study,^41^ automated potentiometric analyzer (the NOVA 8 electrolyte analyzer [NOVA Biomedical, Mississauga, ON, Canada]) in the Canadian study in whole blood,^42^ and Olympus AU 400 Chemistry auto analyzer in India (CLSI C28‐A2).^31^ Pruden et al^46^ demonstrated that RIs observed by photometric method were higher than those observed by flame/atomic absorption methods, though our RI which was obtained by photometric method was lower than other countries. In addition to analytical considerations, gastrointestinal absorption and urinary excretion are known to influence serum magnesium levels.^47^ Therefore, it is possible that differences in renal and gastrointestinal functions of Iranians affect serum magnesium concentrations as a result of genetics. Indeed, the effect of genetics and mutations on hypomagnesaemia has been established previously.^43^

### 4.2 RIs for micro‐minerals

Reference intervals for micro‐minerals of zinc and copper were evaluated. Previous RIs established for zinc are shown in Table 2 for men and women of Tehran (Iran),^28^ Nigeria,^48^ Greek,^49^ and Germany.^29^ Method of zinc measurement was flame atomic absorption spectrometry in studies from Tehran,^28^ Nigeria,^48^ and Greece,^49^ though the method applied by the German group was electrothermal atomic absorption spectrophotometry.^29^ Sex partitioning was reported as necessary in Greek adults and Iranians, in contrast to our results, while age partitioning was not reported by these studies. Ghasemi *et al*. established a RI for zinc using a cohort of 2,632 aged 20–90 years old in Tehran, Iran, in accordance with IFCC guidelines (nonparametric method).^28^ The differences observed between RIs might be related to sample population characteristics, including location, age, and sample size. The various RIs for serum zinc may also be related to the different diet of each population, since direct associations between dietary intake and serum concentration of zinc have been reported previously in cross‐sectional and randomized clinical trials (RCT).^50^, ^51^

To compare our results, the recommended RIs for copper are shown for populations from Nigeria,^48^ Greece,^49^ and Germany^29^ in Table 2. Presented RIs in these studies were significantly wider than that established in the current study. In particular, the established lower reference limit for copper was significantly lower in this study when compared with others. Similar to the parameters described above, these differences may result from the age range, region of life, quality of life, and sample size of the separate studies. In addition, previous cross‐sectional studies have demonstrated that the intake of copper and its serum level is positively associated with each other^50^, ^52^; therefore, the various dietary patterns can influence the reference value distributions of various geographic areas. Furthermore, the method of copper measurement was equal to our study in Nigeria^48^ and Greece^49^ studies, though the method of Germany^29^ study was electrothermal atomic absorption spectrophotometry.

### 4.3 RI for SOD enzyme

Tamai et al^53^ demonstrated that the polymorphism of SOD gene is associated with an increased susceptibility to insulin resistance and type 2 diabetes mellitus. As well, lower SOD activity is considered an independent risk factor for intimal thickening of carotid artery.^54^ Despite its potential clinical indications, few studies have reported RIs for serum SOD. Bogdanska et al^27^ suggested an RI of 0.12–0.33 U/L for SOD enzyme in 111 men aged 18–59 years old from Macedonia. This result is extremely different to our RI of 0.40–4.99 U/L. Higher levels of serum SOD are associated with more amounts of pro‐oxidants that SOD opposes.^19^ The differences observed for SOD enzyme activity level, as a free radical scavenger, might be related to oxidant status,^19^, ^55^ different dietary patterns between populations; as more consumption of anti‐inflammatory diet can lead to decreased oxidative stress in body^56^ and finally lower level of SOD function. Also, the analytical method for measuring SOD activity may influence on various RIs; Bogdanska et al^27^ measured the SOD activity by measuring the inhibitory potential of SOD to the reaction between xanthine/xanthine oxidase and 2‐(4‐iodophenyl)‐3‐(4‐nitrophenol)‐5‐phenyltetrazolium chloride (this reaction generates superoxide radicals); the absorbance of this reaction was monitored at 505 nm by spectrophotometer. The normal distribution was calculated by Gaussian curve.^27^

### 4.4 Strengths and limitations

The strength of our study include the large sample cohort obtained from Mashhad Stroke and Heart Atherosclerotic Disorder (MASHAD) study. It is also the first study to define a RI, by using CLSI Ep28‐A3 guidelines, for calcium, phosphate, magnesium, copper, and SOD enzyme in Iranians. The limitations of this study include: 1) the limited age range (for male of 35–65 years and for females of 37–65 years), 2) sample size variation between parameters, and 3) fewer participants for macro‐minerals in the later age range. In addition, other variables, such as PTH, vitamin D, alkaline phosphatase, Cr, and GFR that may influence mineral concentrations were not explored.

## 5 CONCLUSION

The current study established sex‐ and age‐specific RIs for macro‐minerals (calcium, phosphorus, and magnesium), micro‐minerals (zinc and copper), and SOD enzyme in Iranian males and females aged 35–65 year. These results will be of direct benefit to clinical laboratories across the province and can contribute to improve test result interpretation and more evidence‐based clinical decision‐making in mineral assessment. Implementation is also potentially feasible in other parts of the country, as long as each laboratory performs appropriate verification/validation protocols based on CLSI guidelines.

## CONFLICT OF INTEREST

The authors have no conflict of interest to disclose.

## Data Availability

The data that support the findings of this study are available from the corresponding author upon reasonable request.
